# Making Synthetic 2D Graphene Oxide Nanosheets by Electrochemical Oxidation of Commercial Carbon Fibres

**DOI:** 10.1002/smll.202408972

**Published:** 2025-02-21

**Authors:** Alba Español, Anton Bjurström, Björn Birdsong, Fritjof Nilsson, Annu Pandey, Xiaojun Ren, Rakesh Joshi, Stefano Farris, Richard T Olsson

**Affiliations:** ^1^ Department of Fibre and Polymer Technology, School of Chemical Science and Engineering KTH Royal Institute of Technology Stockholm SE‐106 91 Sweden; ^2^ Wallenberg Initiative Materials Science for Sustainability, Department of Fibre and Polymer Technology KTH Royal Institute of Technology, Stockholm Stockholm SE‐106 91 Sweden; ^3^ NKT HV Cables, Technology Consulting Västerås SE‐721 78 Sweden; ^4^ School of Materials Science and Engineering University of New South Wales Sydney NSW Australia; ^5^ Department of Food, Environmental and Nutritional Sciences (DeFENS) University of Milan via Celoria 2 Milan 20133 Italy

**Keywords:** carbon fibres, electrochemistry, exfoliation, graphite, synthetic graphene oxide

## Abstract

The study demonstrates a scalable and reproducible method for synthesising graphene oxide (GO) nanosheets from commercial carbon fibres derived from carbonised polyacrylonitrile (PAN) polymer. An exfoliation route with nitric acid allows for the preparation of monolayer GO nanosheets with a consistent thickness of 0.9 ± 0.2 nm, identical to the commercially available GO from mined graphite. The GO nanosheets exhibit distinct circular and elliptical shapes, in contrast to the polygonal and sharp‐edged morphology of commercial GO. An extensive evaluation of acidic solutions and electrical potentials identified a narrow processing window critical for obtaining GO nanosheets sized 0.1–1 µm. An unexpectedly low 5% acid concentration was found to be the most effective, providing a balance between efficient exfoliation through synergistic acidic and electrochemical oxidation. The process provides a high yield of 200 mg of GO per gram of carbon fibre. Advanced characterisation using high‐resolution electron and atomic force microscopy (HR‐TEM/SEM/AFM), Raman spectroscopy, X‐ray photoelectron spectroscopy (XPS), and infrared spectroscopy (FTIR) provided detailed insights into the morphology, thickness, surface functionalisation, and chemical composition of the nanosheets. With its high yield, environmentally sound production, and versatility, the synthesised GO offers transformative potential for large‐scale applications, including energy storage, advanced coatings, high‐performance composites, water purification, and electronic devices.

## Graphical abstract

A scalable, sustainable method is presented for synthesising high‐quality graphene oxide (GO) nanosheets from carbonized polyacrylonitrile (PAN) fibres via electrochemical exfoliation in nitric acid. This method produces monolayer GO with consistent thickness (≈0.9 nm) and unique circular and oblong morphology, reducing dependence on natural graphite resources. The process achieves high yields and efficient exfoliation, making it a viable approach for industrial GO production.

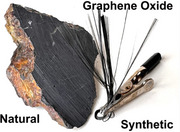

## 1 Introduction

Graphene oxide (GO) is a versatile material with a wide range of applications, spanning biomedical fields, catalysis, and interfaces in multifunctional nanocomposite materials.^[^ ^1^, ^2^, ^3^, ^4^, ^5^ ^]^ In most applications, its large interactive surface area, reaching up to 2800 m^2^/g, makes it highly reactive to its environment.^[^ ^6^, ^7^, ^8^ ^]^ The morphology and nature of the interface makes GO particularly interesting in rapidly growing research areas related to the 2D sheet morphology, which is currently being explored across various applications relying on anisotropic material structures.^[^ ^6^ ^]^ The most common atomic functionalities on the surface of GO are hydroxyl, epoxy‐functional, carbonyl, and carboxyl groups.^[^ ^9^ ^]^ Their ability to interact with their environment, particularly the relative content of reactive epoxide groups, is a topic of research interest due to their ability to be oxidised and functionalised at a lower energy state than other functional groups, such as carboxyl and hydroxyl groups.^[^ ^10^ ^]^ Developing reproducible methods for preparing GO is critical to enabling its exploration in diverse material applications and providing an inexpensive, standardised material for researchers across different fields.

The preparation of GO typically begins with natural graphite, where the 2D sheets are held together primarily by van der Waals forces known as *π–π* stacking due to the sp^2^ hybridisation of carbon atoms. This allows the remaining orbital of the carbon to interact and form a spontaneous stacking of sheets.^[^ ^11^ ^]^ Once the surface is oxidised, the sheets comprise both sp^2^ and sp^3^ carbons, weakening the *π–π* stacking interactions.^[^ ^12^ ^]^ The Hummer method is the most widely used approach for synthesising and surface‐oxidising GO. It provides severe oxidation conditions that allow graphite to be exfoliated into single or multilayer carbon 2D sheets with a range of oxidation states. The proposed mechanism for this oxidation process is a two‐step sequence where sulphate ions in the solution intercalate between the sheets, separating them as oxidation proceeds with potassium permanganate as the oxidation agent.^[^ ^13^, ^14^ ^]^ Although this method has been refined over the years, researchers have sought greener alternatives for GO synthesis, such as performing oxidation at room temperature, reducing the use of acids, or using different oxidisers.^[^ ^1^ ^]^ One promising approach involves using an electrolysis setup to perform the oxidation with less aggressive acidic conditions, although it requires a pre‐treatment step of the graphite.^[^ ^15^ ^]^ While this electrochemical route appears promising for large‐scale synthesis, simplifying the process into a one‐step, continuous method would be advantageous.

A major challenge with any synthesis method using natural graphite as a precursor is the variability in graphite content, which can be as low as 40% in some mineral ores, with the rest consisting of impurities like quartz, plagioclase, or feldspar.^[^ ^16^ ^]^ These impurities complicate the extraction and exfoliation of GO, necessitating additional purification steps to remove adsorbed ions and other contaminants that associate with the large surface area of the GO. Therefore, exploring alternative high‐content carbon sources is worthwhile. Carbon fibres (CFs), produced from the high‐temperature oxidation and graphitisation of polyacrylonitrile (PAN) at 600 and 2000 °C, respectively, CO_2_ offer a pure source of graphitised carbon.^[^ ^17^, ^18^ ^]^ CFs are composed of graphite crystallites and amorphous carbon arranged as a turbostratic structure, where the crystalline planes occasionally slip out of alignment, forming defaults in the carbon matrix and straining the sp^2^ atoms.^[^ ^19^ ^]^

**Figure 1:**
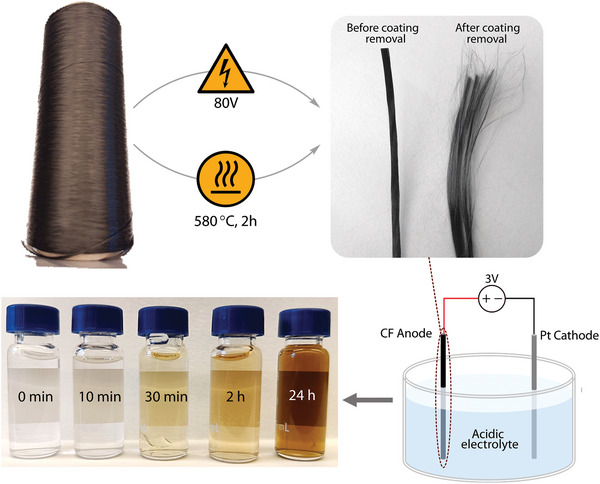
Reaction abstract leading to graphene oxide dispersions in water and evolution of electrolyte colour during electrolysis in 5 wt.% nitric acid under 3 V. The vials display the results from the 1 g scale‐up experiment.

In this work, we report, for the first time, the electrochemical oxidation of CFs derived from PAN as a single‐step method to prepare fully synthetic GO nanosheets. We optimised the specific surface area and reactivity of CFs by testing various acids and their concentrations to determine the most effective conditions for exfoliation. The optimal concentration of nitric acid was established, and the influence of different electrolysis parameters, such as potential and current, on the physical and chemical properties of the resulting GO nanoparticles, was extensively evaluated. This novel synthesis method enables the reproducible preparation of GO without mineral ore contaminants, resulting in 2D GO nanosheets with aspect ratios of up to 500 and thickness ≈ 1 nm. We also investigated two methods for removing the protective polymer coating from commercial CFs: conventional heating at 580 °C for 2 h and shock‐heating at ≈1200 °C for 3 seconds using a high‐voltage transformer, **Figure** **1**. These experiments reveal that the nature of conduction within the CFs significantly influences the electrochemical preparation of GO from commercial carbon fibres.

## 2 Experimental Section

### 2.1 Materials

Carbon fibre tow was purchased from Mitsubishi Chemical Group and consisted of EX‐PAN fibres with a 3000‐filament count (TR 30S 3L). Graphene oxide (GO) was purchased from Angstron Materials (Dayton, USA) as an aqueous suspension with 0.5 wt.% of solid content. According to the manufacturer, the GO flakes had a reported thickness of 1–1.2 nm and a maximum lateral width of 554 nm. Nitric acid (65 wt.%), sulphuric acid, and phosphoric acid were purchased from Sigma‐Aldrich and used as received. MilliQ water (MQw) was used as the aqueous medium (18.2 MΩ, Ph = 7.0).

### 2.2 Removal of Carbon Fibre Coating

Two methods were developed to remove the protective polymer coating from the purchased fibres. The first method subjected the fibres to a direct current (DC) electric field of 80 V m^−1^ for ≈3 s. This method, based on the work by Pourrahimi et al., rapidly heated the fibres to ≈1200 °C, causing the polymer coating to sublimate into gas.^[^ ^20^ ^]^ The fibre strand used in this method was 1.5 meters long and composed of ≈3000 filaments. In the second method, adapted from Pötschke et al., the fibres were heated in an oven at 600 °C for 2 h.^[^ ^21^ ^]^ The fibres were observed by microscopy to confirm fibre uniformity after the treatments (Figure S1, Supporting Information).

### 2.3 Electrochemical Oxidation of Carbon Fibres in Acid Conditions

GO sheets were exfoliated from carbon fibres by electrochemical oxidation. The fibres were submerged in aqueous nitric acid solutions at different concentrations (1, 5, 10, and 50 wt.%), i.e., as electrolytes. The carbon fibres were used as received or with the external protective coating removed via either high voltage treatment or high temperature. A platinum electrode was used as a cathode. The 2‐electrode system was evaluated as a potentiostatic setup at different voltages (2, 3, 4, and 5 V). After 6 h, the suspension was filtered, and the anions in the solution were removed via ion exchange resin. The resulting solutions appeared in a scale of different yellow and brown colours (Figure 1). The reaction was considered finished when the current dropped to 0.00A. The procedure was evaluated for sulphuric, phosphoric, and lactic acid at different concentrations as alternative electrolytes (Table S1, Supporting Information).

### 2.4 Post‐Treatment of the Electrolyte Solution

The obtained coloured solutions containing the exfoliated GO (Figure 1) were processed using different drying methods for optimised drying. In the first method, droplets of the solutions were naturally evaporated under ambient temperature and pressure. The second method consisted of depositing drops of the suspension onto a metallic surface heated at 110 °C, sufficient to evaporate the solvent immediately and limit the Leidenfrost effect. The third method was based on quickly freezing the sample before freeze‐drying the material, as explained by Birdsong et al.^[^ ^22^ ^]^

### 2.5 Characterization

#### 2.5.1 Microscopy

The morphology of the nanoparticles was investigated using a field emission scanning electron microscope (FE‐SEM; Hitachi S‐4800, Japan). The samples were placed on conductive carbon tape, on mica sheets or directly on the aluminium sample holder. Images were taken at an accelerated voltage between 1 and 3 kV and a current of 10 µA. Transmission electron microscopy (TEM) was used to further characterise the morphology, and selected area electron diffraction (SAED) was collected simultaneously to determine the crystal structure using a JEOL JEM‐F200 Multi‐Purpose FEG‐S/TEM. The samples were deposited as droplets on a TEM grid (Ultrathin Carbon Film on Lacey Carbon Support Film, 400 mesh, Copper) from an aqueous solution with exfoliated GO and left overnight for drying.

#### 2.5.2 AFM

To measure the thickness of the nanoparticles, a MultiMode 8 atomic force microscope (AFM) equipped with an RTESP‐150 cantilever (Bruker, Santa Barbara, CA, USA) was used, operated in QNM (Quantitative Nano‐Mechanics) mode under an air atmosphere. The nanoparticles were deposited as 4 µl suspensions with an estimated 0.03 wt.% solid content onto freshly cleaved mica discs and allowed to dry at room temperature until the water completely evaporated.

#### 2.5.3 FTIR Spectroscopy

Infrared spectroscopy was conducted on the freeze‐dried samples using a PerkinElmer Spectrum 100 instrument equipped with an Attenuated Total Reflection (ATR) accessory, a Medium IR detector, and a Specac Golden Gate with a sapphire crystal. The measurements were performed at a scanning rate of 1 cm⁻¹, with a resolution of 4 cm⁻¹, using 32 consecutive scans within the 600 to 4000 cm⁻¹ range.

#### 2.5.4 Raman Spectroscopy

Absorbance was recorded with an I‐Raman Plus 785S portable spectrometer (B&W TEK, Plainsboro, US) with an Argon ion laser at an excitation wavelength of 532 nm at ambient temperature.

#### 2.5.5 Tensile Testing

Tensile testing was performed using an Instron 5944 Universal Testing Machine with a 250N load cell at a constant strain rate of 1 mm min^−1^ until the fibres broke. The fibres were glued to paper to increase friction with the hydraulic grip.

#### 2.5.6 Electrical Measures

The electrical characterisation of the samples included both *I–V* measurements and chronoamperometry, which was carried out using a 2450 Keithley SourceMeter (USA). Chronoamperometry was performed on a single bundle of uncoated carbon fibres (containing 3000 filaments) under 3 V for 2 h. Triplicate I‐V curve measurements were made using carbon fibre as the anode, platinum as the cathode, and silver chloride as the reference electrode.

#### 2.5.7 X‐Ray Photoelectron Spectroscopy (XPS)

XPS measurements were performed using a M‐Probe instrument (Surface Science Instruments, USA) equipped with a monochromatic Al Kα source (1486.6 eV) with a spot size of 200 × 750 µm^2^ using a pass energy of 25 eV, allowing for a resolution of 0.74 eV. The C1s peak level was used as an internal reference for all the samples at 284.6 eV and recorded data was adjusted accordingly. The accuracy of the reported binding energies (BE) can be estimated to be ± 0.2 eV. Wide scan XPS survey spectra were used to gather information on the surface atomic composition of commercial GO sheets and exfoliated GO nanoparticles. High‐resolution spectra of C1s were used to determine the carbon functionalities of commercial and exfoliated GO through deconvolution. The C1s and N1s peak data was imported to OriginPro 2020 version 9.7.0.188 (US) where it was baseline corrected. Subsequently the C1s peak was smoothed using a Savitzky‐Golay filter of second order with 10‐point windows, no filtering was used for N1s as too many features were lost. Gaussian peaks were fitted at expected binding energies and adjusted iteratively until a converging fit.

### 2.6 Statistical Analysis

Experiments were performed thrice per sample to confirm repeatability of the electrolysis process. Particle sizes were determined by measuring the maximum particle length on SEM images of 75 particles per sample (225 total measurements per triplicated sample), and histograms were constructed using ImageJ, National Institutes of Health, Bethesda, Maryland, USA. Data is also presented as mean ± SD. AFM data pre‐processing was analysed using NanoScope Analysis 1.8 software to determine the particle thicknesses, utilising a 1st order polynomial flattening function. The average particle nanosheet thickness values were determined by taking triplicate measurements on nine particles for each sample, resulting in a total of 30 measurements. Data presented is the mean value of the 30 measurements ± SD. FTIR spectroscopy baselines for all curves were obtained using PerkinElmer Spectrum Version 10.5.3 software. FTIR measurements were performed twice for each sample as no outliers were observed. Raman spectra were plotted using a LOESS polynomial regression with a 0.1 span. The reported values of tensile were the average of 3 measurements on 3000 filaments within the single fibres. Deconvolution of XPS data was performed iteratively until a fit with a chi‐square tolerance value of 10^−9^ was reached.

## 3 Results and Discussion

### 3.1 Graphene Oxide Nanosheets Morphology

**Figure 2:**
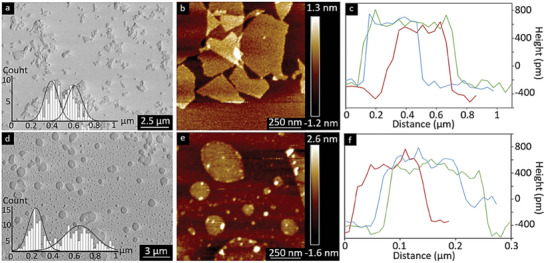
a) SEM Micrographs, b) AFM tapping image, and c)height profile of commercial GO sheets (a–c) after being diluted ten times and deposited on a mica sheet, whereas d–f) show the exfoliated GO sheets. The histograms depict the lateral size distributions using the longest axis for 225 measurement per type of sample.

**Figure** **2** compares the commercial graphene oxide (GO) nanosheets (Figure 2a–c) with those successfully exfoliated from carbon fibres (Figure 2d–f) using a single‐step method that did not require any chemical pre‐treatment of the fibres. The formation of monolayer nanosheets was only observed at an optimised 5 wt.% nitric acid electrolyte and a potential of 3V for the carbon fibres thermally treated at 600 °C (in a conventional high‐temperature oven), see Figure d–f. The effect of acid concentration at different potentials is further discussed in Sections 3.4 to 3.6. From here on, the commercial GO (Figure 2a–c) will be used as a reference for comparison in the study of the morphology and chemistry of the exfoliated GO sheets.

The size distributions of commercial and exfoliated nanosheets are shown as insets in the SEM micrographs and summarised as average values in **Table** **1**. While the commercial GO exhibited a narrower size range of 0.2 to 0.8 µm compared to the exfoliated GO from carbon fibres, which ranged from 0.1 to 1 µm, the average lateral sizes were nearly identical. The thickness of the electrochemically synthesised GO monolayer sheets from carbon fibres was 0.9 (± 0.2) nm, consisting of single 2D layers of carbon and oxygen functional groups. This value aligns with the reported literature range of 0.40 to 1.13 nm (see Figure 2c,f). However, it is unclear how the similarity in the lateral dimension of the CF exfoliated GO with the commercial GO can be explained.

**Table 1:** Morphological properties of commercial and exfoliated GO.

Materials	Average Thickness [nm]^a)^	Average Dimension [nm]^a)^
Commercial GO	0.9 ± 0.1	513 ± 190
Exfoliated GO	0.9 ± 0.2	511 ± 287

^a)^ Average particle thickness was determined from multiple measurements per sample using AFM, while the lateral dimensions were determined from 75 measurements using ImageJ from SEM images per sample. The sample measurements for the exfoliated GO were made as triplicates (225 total measurements).

The commercial GO sheets (Figure 2a,b), derived from natural graphite, exhibit a polygonal shape with straight edges and sharp corners, resembling the morphology of exfoliated graphite sheets reported by Panzarasa et al. in 2019.^[^ ^23^ ^]^ In contrast, the exfoliated sheets have a circular or elliptical shape with no sharp edges, as shown in Figure 2d,e, which aligns more closely with the platelet model described by Love‐Baker et al. in 2024, where the crystallites appear cylindrical.^[^ ^24^, ^25^ ^]^ It is suggested that the absence of a strict crystalline lattice in the carbon fibres resulted from a less uniform oxidation pattern, producing GO sheets that lack the sharp edges characteristic of natural graphite‐derived GO.

### 3.2 Chemical Analysis of GO Sheets

**Table** **2** presents the atomic weight percentages of carbon (C), oxygen (O), and nitrogen (N) in the commercial and the exfoliated GO nanosheets, as determined by Energy Dispersive Spectroscopy (EDS).

**Table 2:** The EDS analysis of commercial GO and exfoliated GO nanosheets. The measurements were performed on an agglomerated sample (0.7mm) using a 4 keV energy source.

Chemical Formula	C [wt.%]	O [wt.%]	N [wt.%]	Total (%)
Commercial GO	53.5	46.5	0.0	100
Exfoliated GO	26.9	53.0	20.1	100

The commercial GO exhibited two predominant peaks corresponding to carbon and oxygen, with the nanosheets consisting of ≈ 50% oxygen (46.5 wt.%), meaning the material was highly oxidised. Typical values for the relative carbon/oxygen ratio in GO have been reported to range between 4.9 and 21.2.^[^ ^26^ ^]^ The EDS measurements of the exfoliated GO indicated that the sample was composed of even more oxygen than carbon. This substantial amount of oxygen was linked to the presence of nitrate residues, which may be expected from the electrochemically obtained GO using nitric acid as an oxidant.^[^ ^15^ ^]^ However, it was not possible to confirm whether the surface‐absorbed nitric acid residues were covalently bonded or merely physically absorbed on the GO nanosheets via this method.

**Figure 3:**
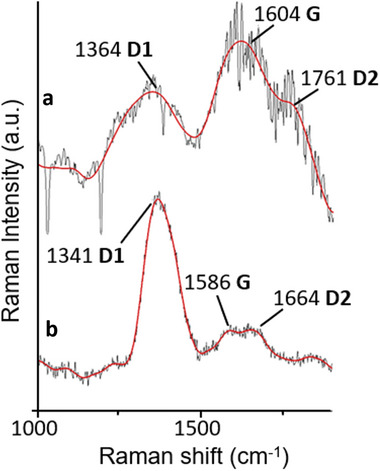
Raman Spectra of freeze‐dried a) commercial GO and b) exfoliated GO nanosheets. The G‐band is the in‐plane vibration of sp^2^‐bonded carbon atoms in the crystal lattice, whereas the D‐band is associated with structural defects.

**Figure** **3** shows the Raman spectra of freeze‐dried samples for the commercial and exfoliated GO. Both samples exhibit the characteristic D and G bands of graphene oxide, as reported by Stankovich et al.^[^ ^27^ ^]^ The spectra show that the commercial GO showed a D band at 1364 cm^−1^ and a G band at 1604 cm^−1^, see Figure 3a. Notably, the intensity of the D band peak is lower than that of the G band peak, whereas the opposite behaviour was observed for the exfoliated GO (Figure 3b), where the G band (1586 cm^−1^) is significantly smaller in intensity compared to the D (1341 cm^−1^) band peak. These results are consistent with more extensive oxidation (and a greater degree of structural disorder) of the electrochemically exfoliated GO than the commercial GO, confirming the results observed by EDS analysis. Overall, the D1, G, and D2 bands were shifted ≈20 cm^−1^ for the exfoliated GO to lower, 1364–1341 cm⁻¹, 1604–1586 cm⁻¹, and 1761–1664 cm⁻¹, respectively. This significant red shift was previously observed for covalently functionalised graphene and graphene oxides and is consistent with the electrochemically exfoliated GO being a highly oxidised material covalently functionalised by nitrogen oxides.^[^ ^28^, ^29^ ^]^ Both spectra show light shouldering on the G peak, which could be identified as a D2 peak, suggesting that both types of GO have structural in‐plane imperfections that do not completely disrupt the sp^2^ hybridised carbon network.

X‐ray Photoelectron Spectroscopy (XPS) was employed to investigate the differences in chemical surface composition between commercial and exfoliated graphene oxide (GO). **Table** **3** presents the atomic composition of the two samples, derived from the wide‐scan XPS survey spectra.

**Table 3:** Elemental surface analysis of freeze‐dried commercial GO and exfoliated GO nanosheets determined by XPS. C/O is the carbon‐to‐oxygen ratio calculated from the respective measured amounts.

Chemical Formula	C [%]	O [%]	N [%]	Other^a)^ [%]	C/O
Commercial GO	86.2	10.4	0.0	3.4	8.3
Exfoliated GO	62.5	23.7	8.8	4.9	2.6

^a)^ Traces of silicon and sulphur.

The XPS data confirmed a higher degree of oxidation in the exfoliated GO compared to the commercial GO, with an increased oxygen content (23.7% vs 10.4%) and the presence of nitrogen (8.8%). The discrepancies between the atomic compositions observed in XPS and those obtained from EDS was attributed to the differing sensitivities of the techniques: XPS is more surface‐sensitive and provides a more accurate analysis of surface chemical composition, whereas EDS is bulk‐sensitive and underestimates the relative carbon content in GO. Nonetheless, the relative ratio of oxygen to nitrogen exhibited a deviation of only ≈2% between EDS and XPS measurements, with both values aligning with previously reported data.^[^ ^30^ ^]^ The presence of nitrogen in the exfoliated GO was attributed to the exfoliation process in nitric acid, distinguishing it chemically from the commercial GO used for comparison.

**Figure 4:**
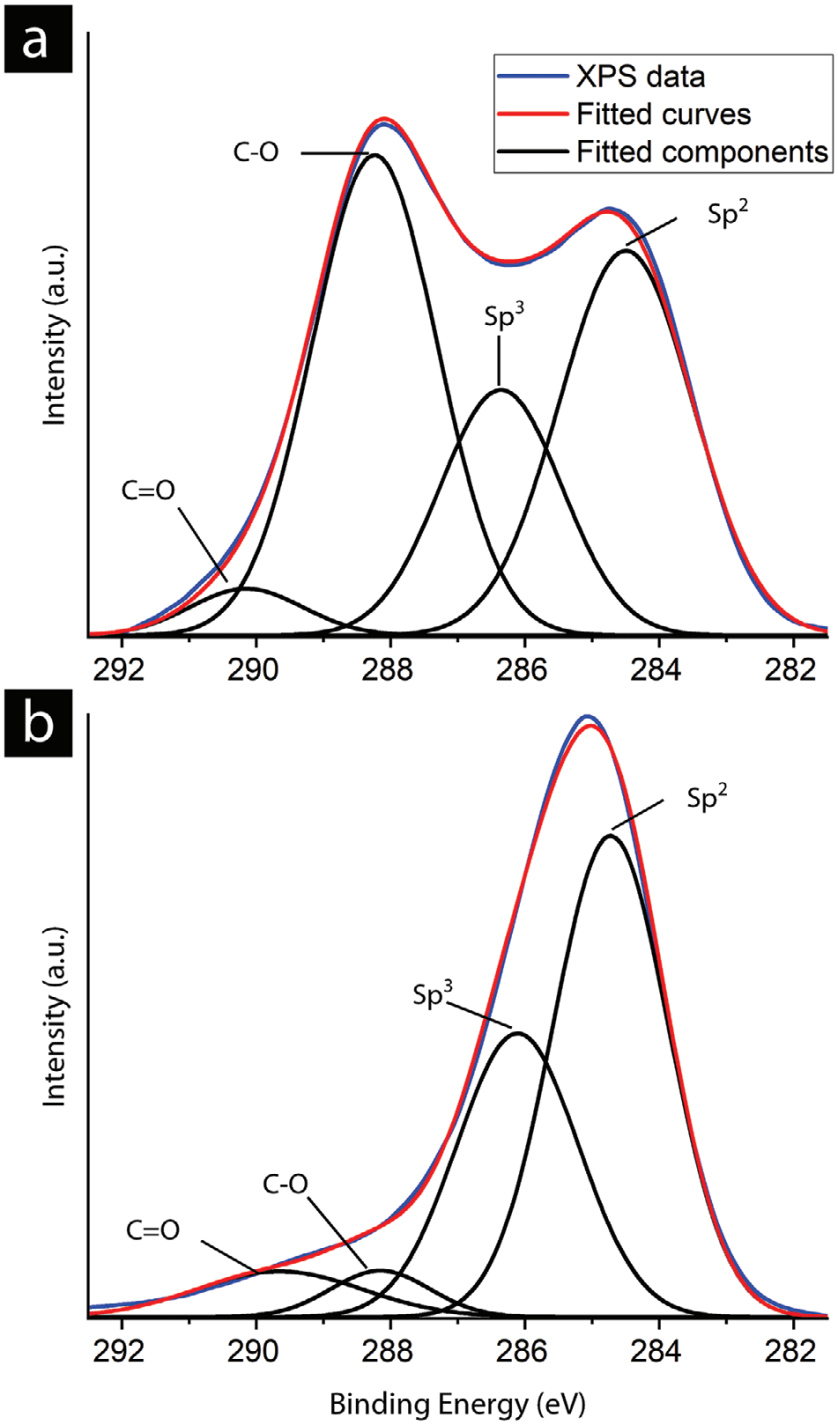
High‐resolution C1s XPS spectra of freeze‐dried a) commercial GO and b) exfoliated GO nanosheets deconvoluted for the carbon functionalities.

**Figure** **4** shows the high‐resolution C1s XPS deconvoluted spectra of the freeze‐dried samples, revealing two different patterns. Commercial GO showed a saddle‐like pattern, whereas the exfoliated GO showed a pattern with a distinct peak. More specifically, commercial GO exhibited the usual saddle‐like pattern characteristic of oxidised carbon structures.^[^ ^31^ ^]^ The deconvolution suggests the presence of four different peaks, showing the sp^2^ (284.6 eV) and sp^3^ (286 eV) hybridisation of carbon, as well as the carbon‐oxygen bonds, single (≈288 eV) and double (≈290 eV), present in the material, corresponding to previously reported values.^[^ ^32^, ^33^ ^]^ This measurement allows the proper quantification of sp^2^ and sp^3^ carbons, indicating that the sample did not undergo severe damage with the measurements. In commercial GO, the sp^2^/sp^3^ ratio is 1.71; in the exfoliated GO, a similar ratio of 1.6 is observed. However, a notably lower amount of carbon‐oxygen bonds is observed in the exfoliated sample, pointing to a comparatively lower amount of directly bonded oxygen in the form of hydroxyl, epoxide, ether, or carboxyl groups compared to the commercial sample. The high oxygen content for the exfoliated GO is instead related to the presence of surface‐bonded nitrogen.

The N1s XPS spectra (see Figure S2, Supporting Information) show a pattern that can be deconvoluted into three major peaks corresponding in the first two cases to nitrogen oxides, C─NO_x_ (407.5 and 409 eV) and for the last case (402.7 eV) it would be contributed by amines and the C─N bonds.^[^ ^34^, ^35^, ^36^ ^]^ The minor peak (404 eV) would also show for nitro groups, further pointing to this conclusion. The presence of nitrogen determined by XPS proves that the synthesis of GO in the presence of nitric acid under an electric field boosted the formation of new covalent N‐based bonds. It can be concluded that the GO obtained from this synthesis is a 2D oxygen‐nitrogen functionalised material.

### 3.3 Effect of Voltage on Graphene Oxide Materials

**Figure 5:**
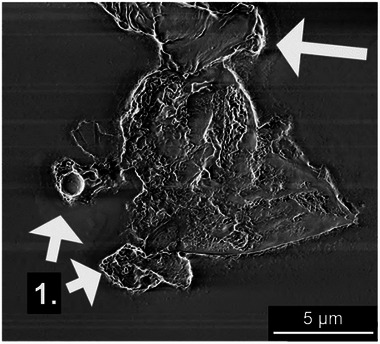
SEM micrograph of oxidised carbon particles fragmented at 5V in 5wt.% nitric acid and deposited on a mica sheet. The particle edges (1) show curling of the material previously reported as due to strong oxidation.

**Figure 6:**
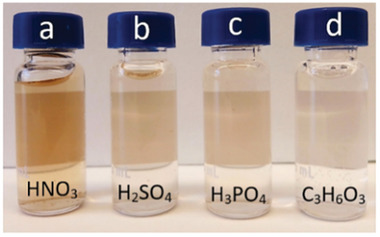
Solutions made under 5 volts during 4 h in 5 wt.% a) nitric acid, b) sulphuric acid, c) phosphoric acid, and d) lactic acid. As a reference, one experiment was conducted in water with sodium chloride, and it was validated that no reaction was observed in the absence of the acid component.

**Figure** **5** illustrates the effect of performing the exfoliation at an elevated potential of 5 volts applied to the carbon fibre (CF), a method previously reported for the synthesis of GO via electrochemical oxidation of expanded natural graphite.^[^ ^15^ ^]^ Compared to the 3‐volt potential successfully employed in Section 3.2, the higher voltage resulted in a more intense colouration of the GO solution, forming significantly faster (≈2 h), as shown in **Figure** **6**. However, the increased potential also accelerated the consumption of carbon fibres, as seen in Figure S3 (Supporting Information), indicating a more aggressive reaction where the higher voltage caused uneven exfoliation and partial fragmentation of the carbon fibres (Figure 5).

The curled‐up sheet‐edge sections observed in the exfoliated samples have previously been linked to severe oxidation of graphite into GO.^[^ ^37^ ^]^ Moreover, the rapid processing at higher voltages negatively impacted the formation of monolayer nanosheets. Specifically, at 4 and 5 volts, monolayer GO sheets did not form, and the resulting exfoliated sheets were thicker. At an exfoliation potential of 4 volts, the particles retained a similar disk‐like morphology as those obtained at 3 volts but with a smaller average size of 150 nm. Atomic Force Microscopy (AFM) measurements revealed an average thickness of 3.1 nm (Figure S4, Supporting Information), with most particles being ≈ 4 nm thick. This indicates that most of the particles produced at this voltage were multilayer graphite oxide, with only a limited presence of monolayer graphene oxide. The fibres only started decomposing at voltages higher than 2.8 volts, which was the lower potential boundary value for the reactions to occur.

### 3.4 Effect of Acid Nature on the Exfoliation of Graphene Oxide Nanosheets

Four different acids were evaluated as electrolytes for the exfoliation of GO to compare their influence on the morphology and functionality of the graphene oxide sheets. The acid concentrations used ranged from 1–50 wt.% for nitric acid, 5–98 wt.% for sulphuric acid, 5–10 wt.% for phosphoric acid, and 5 wt.% for lactic acid, and the experiments were conducted at potentials of 3, 4, and 5 volts. The acids and the conditions are listed in Table S1 (Supporting Information). Among all the exfoliation reactions, the nitric acid electrolyte always exhibited a higher reactivity, and the solutions appeared more coloured after the same duration of electrolysis for the different acids, see Figure 6.

It was noted that increasing the concentration of acid beyond 5 wt.% in solution resulted in a lower reactivity, regardless of the type of acid used. It was, therefore, suggested that the ability to exfoliate carbon nanosheets was not due to the pH of the electrolyte but could be linked to the ion size of the acid. This was further validated for the sulphuric acid, which was used at 98 wt.% concentration, showing no reactivity or signs of exfoliation. **Table** **4** lists the various counter ions used along with their properties, illustrating the relationship between ion size and reactivity. The results indicate that smaller counter ions are more effective in facilitating exfoliation.^[^ ^27^ ^]^

**Table 4:** Counter ion properties.

Counter Ion	NO_3_^−^	SO_4_^2−^	PO_4_^3−^	C_3_H_5_O_3_^−^
Size (pm)	179	258	238	600
pKa_1,2,3_	−1.4	−3.0/2.0	2.2/7.2/12.4	3.9

The ion sizes can be compared to the distance between adjacent carbon layers in crystalline graphite with AB‐stacking, which is ≈ 335 pm.^[^ ^38^, ^39^, ^40^ ^]^ The size of the 179 pm nitrate ion stands out as the smallest among the evaluated while also resulting from the complete dissociation from its conjugated, very strong, HNO₃ acid with pKa of ≈−1.4. This allows it to penetrate the CF structure effectively and exfoliate the GO from graphite as simultaneous water splitting occurred at ≈ 2.34 volts (see Figure S5, Supporting Information). In contrast, the larger sulphate ion (258 pm) associated with H_2_SO_4_ (pK_a2_ of HSO₄⁻ = 2.0) is more ineffective in diffusing into the CF graphite. The smaller acidity (greater pKa) and the larger size of the counter ions for weaker acids (phosphoric and lactic acid) did not allow for exfoliation to occur. Previously, it was reported that electrolyte is intercalated between the graphene sheets in the anode, where it is responsible for the exfoliation of the sheets.^[^ ^41^ ^]^ It was herein observed that carrying out the reactions under lower concentrations than 5 wt.% nitric acid yielded no results even after 4 h of reaction time. Therefore, each acid's reactivity was qualitatively evaluated by the time when colouring appeared in the solution. When the smaller nitrate ions were used, the solution was markedly coloured after merely 10 min of reaction. This can be seen in Figure 1, which shows the colouration of the nitric‐acid exfoliated GO after 10 min, 30 min, 2 h, 10 h, and 24 h reaction. In comparison, phosphoric acid and sulphuric acid solutions only showed colour after 1 h 30 min and 2 h 10 min, respectively. Due to the significantly more effective exfoliation using the nitric acid and the counterintuitive result that a higher nitrate ion concentration did not further accelerate the reactions, the exfoliated GO for the different nitric acid concentrations were investigated by infrared spectroscopy.

### 3.5 Effect of CF Preparation Before the Exfoliation of Graphene Oxide Nanosheets

**Figure 7:**
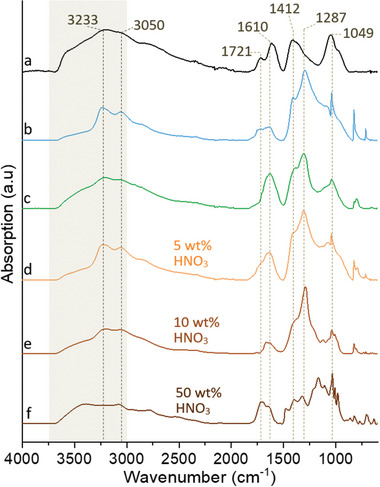
FTIR‐spectra of freeze‐dried a) commercial GO as reference), GO obtained under 3 volts and 5 wt.% nitric acid from b) coated carbon fibres, c) fibres with shock‐removed coating, and d) fibres with oven‐removed coating. d–f) show the influence of different nitric acid concentrations (5, 10, and 50 wt.%) on the electrolysis process.

**Figure** **7** shows the commercial GO sheets for reference (a), and the spectra of the GO exfoliated directly from the epoxy‐coated CF (b), the 3s high‐voltage treated CF (c), and the 2h 600 °C oven‐heated CF (d), using 5 wt.% nitric acid.

The commercial reference GO exhibited four distinct peaks in the range of 1800–900 cm⁻¹, corresponding to different functional groups. These peaks were assigned to single carbon‐oxygen bonds at 1049 cm⁻¹, double carbon‐oxygen bonds at 1721 cm⁻¹, carboxyl stretching at 1412 cm⁻¹, and conjugated carbon‐carbon bonds at 1610 cm⁻¹.^[^ ^42^ ^]^ Additionally, a broad shoulder observed in the 3500–3000 cm⁻¹ range was attributed to ‐OH groups and adsorbed water, resulting from the GO's exposure to air.^[^ ^43^ ^]^ For the exfoliated GO samples, the peaks closely matched those of the reference GO, except for two additional shoulder peaks in the 3000–3500 cm⁻¹ region, as seen in Figure 7b–d. These shoulders were attributed to single nitrogen‐hydrogen at 3050 cm⁻¹ and sp^2^ hybridised carbon at 3233 cm⁻¹. The presence of N‐H peaks is mainly attributed to nitric acid residue in the dried sample. The intensity of the shoulder at 3233 cm⁻¹ decreased with increasing acid concentration and was almost absent in the high‐voltage‐treated carbon fibre (CF) sample (Figure 7c). This phenomenon suggests that the formation of less oxidised nanosheets, which contain more sp^2^ hybridised carbon, reduces the presence of ‐OH groups and adsorbed water, leading to the diminished shoulder peak. In contrast, more oxidised forms of GO (Figure 7c,e,f) contained a higher concentration of hydroxyl groups, which readily associate with water, making these peaks more prominent.^[^ ^44^ ^]^

The more oxidised surface was also synonymous with a smaller presence of carboxyl groups for the nanosheets from the 3s high‐voltage treated CF (Figure 7c), as notable from the missing peak at 1721 cm^−1^. At the same time, an increasing concentration of nitric acid up to 50 wt.% accordingly did not only cover the sp^2^ hybridised carbon at 3233 cm^−1^ and sp^2^ hybridised carbon at 3050 cm^−1^, respectively, but also allowed for a relatively greater amount of carboxyl groups (1721 cm^−1^).

The IR results highlight the difficulties in drawing conclusive statements on the relative presence of functional groups as correlated toward sp^2^ hybridised carbon content in the exfoliated GO. However, with the combined observations, it was possible to conclude that the 2‐h thermal exposure to 600 °C was the most promising route to obtain the pristine GO nanosheets for further optimisation. The choice to abandon the rapid 3s high‐voltage treatment at an estimated 1200 °C was not only based on the apparent higher degree of fibre oxidation and limited IR confirmation of the sp^2^ hybridised carbon at 3050 cm^−1^ but also on the fact that it was in many experiments practically challenging to exfoliate GO from the high‐voltage treated fibres effectively. This was partly explained by electrical contact problems with the nitric acid solution due to fibre breakage occurring along the fibres during the high‐voltage treatment due to locally excessively heated regions. This was not a problem for the fibres evenly heated at 600 °C for 2 h.

### 3.6 Electrical CF Current Over the Time for the Entire Exfoliation Duration of the Nanosheets

**Figure 8:**
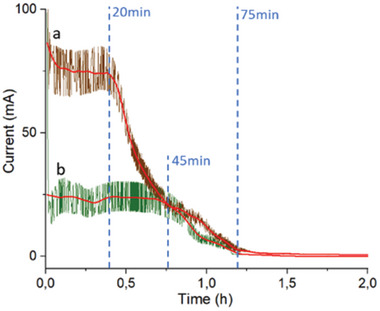
Current (at 3 volts) over time during the 5 wt.% nitric‐acid exfoliation of the GO for a) thermally treated fibres at 600 °C and b) for high‐voltage treated fibres.

**Figure** **8** shows the conductive properties of the fibres over time during the entire exfoliation reaction for the two cases, that is, the more uniformly 600 °C heat‐treated fibres (Figure 8a) as compared to the 3s high‐voltage treated fibres (1200 °C), Figure b.

**Figure 9:**
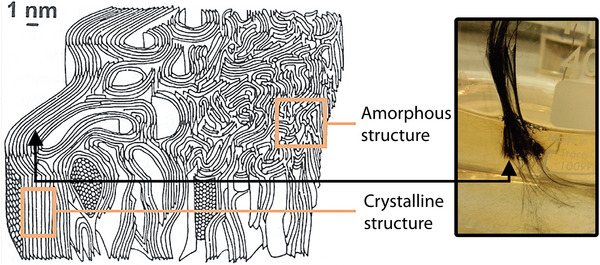
Shows the mixed internal phase structures of a commercial carbon fibre turbostratic phase, including crystallites and amorphous carbon. Adapted from Bennett et al.^[^ ^46^ ^]^

The effective exfoliation reaction lasted for ≈1 h (75 min), corresponding to the period of efficient electron transfer within the crystalline layered sections of the carbon fibres (CF), depicted in **Figure** **9**. Despite the mechanical properties of the fibres being more compromised after the 2‐h heat treatment at 600 °C (see **Table** **5**), this treatment resulted in better and more consistent exfoliation compared to the 3s high‐voltage treatment at 1200 °C. It is suggested that the more uniform thermal treatment at 600 °C facilitated a gradual exfoliation process, which was not achievable with the short high‐voltage treatment. The latter caused large fragments of material to detach, to some extent hindering the formation of GO monolayers. A similar effect was observed with increasing nitric acid concentrations. As the concentration increased from 5 to 10 wt.% and then to 50 wt.%, the monolayer exfoliation was completely absent at the highest concentration. Instead, only large chunks of carbon material detached from the fibres, further demonstrating that excessively rapid oxidation or aggressive conditions lead to undesired fragmentation rather than controlled exfoliation.

**Table 5:** Tensile testing performed on carbon fibres under a constant 1 mm min^−1^ strain.

Fibres	Coated [Ref.]	High‐voltage treated [3 s, 1200 °C]	Thermally treated [2 h. 600 °C]
Tensile strength (GPa)	3.17 ± 0.40	1.85 ± 0.06	1.12 ± 0.23

Previously, it was reported that the internal structure of the carbon fibres can be depicted according to Figure 9, which stems from a gradual oxidation of the polyacrylonitrile polymer at ca 200 to 300 °C, followed by a carbonisation step in an inert environment at 1000 to 1700 °C.^[^ ^45^ ^]^ An additional graphitisation is commonly carried out at 2500 to 3000 °C under an inert atmosphere, determining the conductive nature and occurrence of the sp^2^ hybridised planes, providing a significantly enhanced conductivity post graphitisation reaction. The Figure 9 inset shows the depleted fibres still holding together after the exfoliation reaction. At this point, the exfoliation was completed, and a critical amount of sp^2^ hybridised nanosheets had been exfoliated, resulting in the fibres no longer being electrically conducting under these conditions (Figure 8).

### 3.7 Drying GO Nanosheets into Particle Morphologies

**Figure 10:**
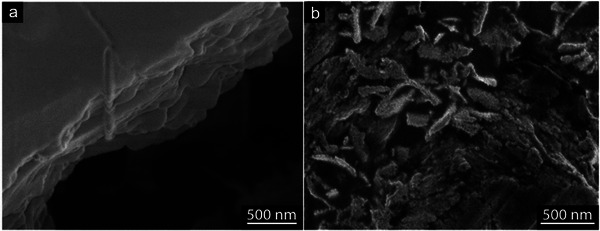
SEM Micrograph of graphene oxide made under 3 V and 5 wt.% nitric acid solution after a) being left to dry in ambient conditions and b) after diluting the sample and depositing 4 µL suspension on the aluminium sample holder.

The exfoliated graphene oxide showed a strong tendency to agglomerate, and different methods have previously been tested to achieve non‐agglomerated particles.^[^ ^10^ ^]^ Air drying of the GO suspension under ambient conditions resulted in a cracked film. The GO nanosheets could, however, be observed as stacked sheets in a book‐like structure on the edges of the cracked film, see **Figure** **10**a. When the water‐suspended GO was diluted ten times and deposited as a less concentrated solution directly on the aluminium microscopy stub, the agglomeration was limited, and it was reasoned that removing the solvent rapidly would not allow the sheets to have sufficient time to agglomerate. To further limit the agglomeration, the SEM sample stub was heated to 110 °C. The material was added dropwise, evaporating the solvent almost instantaneously when reaching contact with the aluminium metal surface. This method was, however, not sufficiently efficient, and the sheets still formed a cracked film, which can be seen in Figure 10b. The observed challenges involved in the formation of a uniform GO film limited the possibility of accurately obtaining film resistance with the 4‐wire collinear probe, connected to the Keithley 2450 Source Meter.^[^ ^31^ ^]^

### 3.8 Crystalline Structure of Synthetic GO Nanosheets

**Figure 11:**
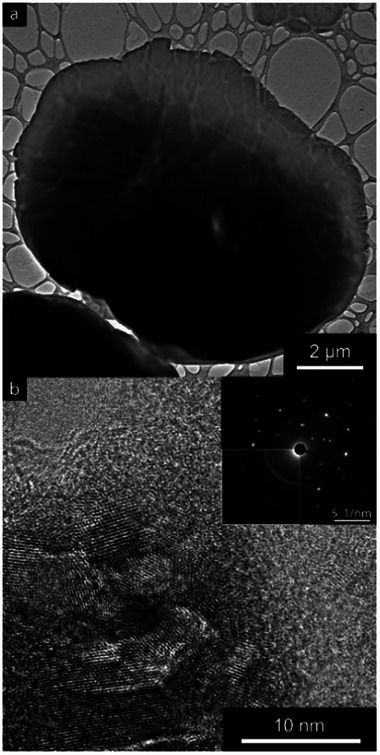
HR‐TEM micrographs of graphene oxide sheets highlighting a) the stacked nature and b) the crystalline structure of the sheets with an inlet of the selected area electron diffraction pattern and a polycrystalline commercial graphene oxide in the bottom left quadrant for comparison.

**Figure** **11**a shows an HR‐TEM micrograph of one of the largest observed graphene oxide sheets, with a cross‐sectional 2D diameter of ca 10 µm. The sheet's size is much bigger than that of the nanosheets observed by AFM, see Figure 2, indicating that it did not only consist of one but several agglomerated and stacked sheets. The limited transmission in some areas of the sheet also suggested that the sheet was of non‐uniform thickness, which further points to this conclusion. The observed fringe patterns of the individual sheets are apparent in the magnified areas of the larger sheet in Figure 11b. The deviating directions of the patterns suggest that multiple crystal planes are present in the magnified regions. The multiple planes can also be seen in the transmission diffraction pattern of the inset in Figure 11b. The nature of the diffraction pattern resembles neither a single crystal nor a polycrystalline graphene oxide. A single crystal would show only a repeating pattern with a fixed distance between the spots. In contrast, a polycrystalline structure would show a pattern of solid rings, similar to the one in the last quadrant of the inset image that belongs to the commercial graphene‐oxide used as a reference in this article. This further points to the interpretation of the presence of several planes of crystals within the larger sheet in Figure 11a.

### 3.9 Modeling and Data Interpretation

The experimental data suggests a complex interplay of factors influencing the exfoliation reaction, particularly the selective penetration of nitrate ions at specific concentrations and potentials, which is related to their size under the given acidic and temperature conditions. This interaction resulted in a relatively high yield (≈20 wt.%, see Section 3.8) and necessitated further modelling to understand the exfoliation process fully.

**Figure 12:**
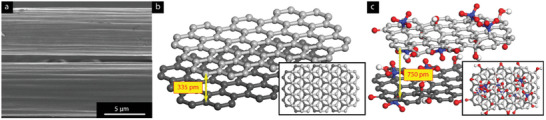
a) SEM Micrograph of HV‐treated fibre before (top) and after exfoliation (bottom), b) modeled crystalline graphite/graphene with AB‐stacking, c), modelled synthetic GO with oxygen content corresponding to XPS‐data (Table 3).

**Figure** **12**a shows SEM images of the carbon fibre (CF) surface before (top) and after (bottom) exfoliation. Despite the exfoliation process, the CF surface appeared visually unaffected and non‐porous at a macroscopic level, indicating that the exfoliated GO sheets were removed without significantly altering the surface morphology. This suggests that the reaction proceeded as a continuous “peeling” mechanism, predominantly occurring during the first 45 min of the reaction (see Figure 8). The radial symmetry of the exfoliated fibre structure, consisting of turbostratic crystalline and amorphous regions with sp^2^‐hybridised carbon sheets oriented parallel to the fibre surface, aligns with the proposed structure in Figure 9. As oxidation progressed, the radial removal of un‐oxidised crystalline layers gradually reduced the fibre's conductivity, which corresponded to the decreasing current measured at 3V over time (Figure 8). After 45–75 min, the reaction could no longer proceed due to the formation of a non‐conducting fibre interface in contact with the acidic solution. Figure 12b illustrates two adjacent sp^2^‐hybridised carbon sheets within a classical graphite structure with AB‐stacking (inset), having an interplanar distance of ≈335 pm, where the van der Waals radii of the carbon atoms are in contact.^[^ ^40^ ^]^ This structure is held together by π‐π stacking interactions and other van der Waals forces.^[^ ^47^, ^48^ ^]^ When ions and small molecules like oxygen and water penetrate the interlayer spaces, the forces binding the layers weaken, and the layers are gradually mechanically forced apart, with the intercalating species acting as wedges. When the penetrant molecules attach to the carbon surfaces, such as through oxidation, the interlayer distance is semi‐permanently increased, facilitating the penetration of additional small molecules. This process leads to the expansion of the graphite layers and subsequent exfoliation of the graphene oxide sheets, as previously reported.^[^ ^49^ ^]^ The smaller molecules and ions can intercalate and diffuse more easily between the layers compared to larger penetrants. This explains why the smallest counter ion, nitrate (NO₃⁻), induced the fastest exfoliation reactions, while the largest ion, lactate (C₃H₅O₃⁻), showed no effect, as its size (600 pm) exceeds the interplanar distance of graphite layers (335 pm) (Table 4). Figure 12c visualises the corresponding layers of electrochemically exfoliated GO, with an oxygen fraction consistent with the XPS data (Table 3). Upon oxidation, the interplanar distance in the crystalline regions increased to ≈750 pm, reflecting the substantial expansion due to the incorporation of oxygen functional groups.

The thickness, density, and interlayer distance of oxidised graphite sheets vary more significantly than those of non‐oxidised graphite. The success of exfoliation with nitrate ions is likely due to their small size relative to the graphite interlayer distance, allowing them to diffuse effectively between the carbon layers. The temperature effect on the process, specifically in terms of graphite layer expansion (<0.25%,^[^ ^50^ ^]^) is minimal for acid penetration but likely affects the diffusivity and oxidative nature of the acids. This suggests that other acids with counter ion sizes smaller than 300 pm may also effectively promote exfoliation at higher temperatures.

### 3.10 Upscale of GO Exfoliation

**Figure 13:**
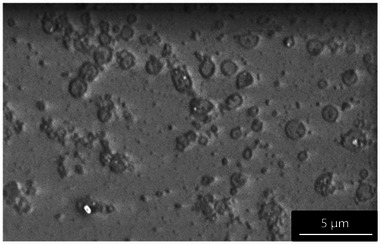
SEM micrograph of graphene oxide nanosheets made at 3 volts and 5 wt.% nitric acid solution, deposited on a mica sheet from 1.0 grams of thermally uncoated carbon fibres.

From the prior 3000 filament investigation, an attempt was made to scale up the exfoliation reaction to 63 000 filaments, ca 1 g of total carbon fibre. The 63000‐filament setup was left overnight for exfoliation, and a deeply coloured solution was obtained after 24 h, see Figure 1. The results from this large‐scale preparation showed that the monolayer GO nanosheet fabrication was extensively scalable, as observed by SEM, see **Figure** **13**.

The corresponding AFM measurements (Figure S6, Supporting Information) showed a thickness identical to the thickness measurements obtained for the GO prepared in smaller amounts (1 ± 0.2 nm). In contrast, the SEM micrographs showed a similar size and size distribution, 561 ± 182 nm, see Figure 13. The upscaled reaction allowed for accurate measurements of the yield of the reaction, and the yield from a total mass of 1 gram was 200 mg per gram of fibre used. SEM observations show a vast majority of graphene oxide nanosheets, with some agglomerated spherical clustersoriginating from the drying conditions, as well as some fibre residue from the electrolysis process (see Figure S7, Supporting Information). Therefore, it is concluded that 20% of the fibre is consumed to produce nanosheets and other oxidised carbon forms, proving the viability of the scaling up of the process. Isolation of the nanosheets and optimisation of the process is therefore a matter of great scientific interest and need to be further explored.

## 4 Conclusion

This study represents a scalable and sustainable production of synthetic graphene oxide (GO) nanosheets, utilising high‐purity carbon fibres derived from carbonised polyacrylonitrile (PAN) as a feedstock. By employing an electrochemical exfoliation process under optimised acidic conditions, the method successfully generates high‐quality monolayer GO nanosheets, ≈600 nm in size, with properties comparable to commercially available GO and an identical thickness of 0.9 ± 0.2 nm. The critical role of pre‐treatment, particularly the thermal removal of protective polymer coatings, was of key importance for the processing outcome. Limitations in the effective processing duration have been identified through detailed experimentation, showing that exfoliation reaches peak efficiency within a 45‐min window. This insight paves the way for continuous processing, wherein carbon fibres can be systematically pulled through the extraction medium and replaced with new fibres, facilitating uninterrupted GO production. The comprehensive evaluation of different acid concentration and potential ranges was used to identify a narrow yet highly efficient processing window, at particularly low concentration of nitric acid. This window enabled precise control over nanosheet morphology and size distribution. The process demonstrates exceptional scalability, achieving a yield of 200 mg of GO per gram of fibre, offering clear potential for large‐scale industrial applications. The incorporation of nitrogen residues in the synthesised GO offers functionalisation possibilities that warrant further investigation to better understand its potential applications associated with bandgap modulation.^[^ ^51^ ^]^ However, the uniform thickness and high surface area also make it interesting within energy related material systems, such as supercapacitors, batteries and as dielectric material in high voltage applications.^[^ ^52^, ^53^ ^]^ Additionally, incorporating nitrogen residues into the synthesised GO introduces active sites, significantly enhancing its catalytic potential, particularly for oxygen reduction reactions (ORRs). Nitrogen doping modifies the electronic structure, facilitating electron transfer and improving the material's reactivity in catalytic applications. Furthermore, functional groups introduced during synthesis will alter the material's adsorption capabilities, positioning it as a candidate for water purification technologies, including the removal of heavy metals and organic contaminants.^[^ ^54^, ^55^ ^]^ The significance of the method is heightened by the European Union's recent classification of natural graphite as a critical raw material, given its limited availability and increasing demand across industries such as electronics, and energy storage, as the intrinsic electrical properties and its possibilities for more extensive bandgap modulation have been established. The demonstrated approach paves the way for a greener, more efficient manufacturing processes while aligning with global efforts to mitigate resource scarcity by reducing dependence on mined graphite. The further exploration of a range of conductive carbon sources is foreseen, including the use of completely renewable and more environmental conscious feedstocks such as lignocellulose‐derived carbon fibres from wood, or other carbonized renewable biomaterials, in steps toward achieving fully environmentally conscious GO manufacturing.

## Conflict of Interest

The authors declare no conflict of interest.

## Author Contributions

A.E. carried out the laboratory experiments and performed most characterisation work, including FE‐SEM, AFM, Raman, FTIR, mechanical and electrical testing with support from A.B., B.K.B., and A.P. The XPS measurements were organised by S.F. and reviewed and interpreted by A.E., A.B., and S.F. The HR‐TEM was performed by X.R. and R.J. and interpreted by X.R. and R.J together with A.B. and R.T.O. The molecular modelling was performed by F.N. and reviewed by A.B., F.N., and R.T.O. The initial version of the manuscript was written by A.E. and R.T.O., and critically reviewed by A.B. and R.T.O. All authors finally reviewed and commented on the manuscript and agreed on its content prior to submission. The concept of utilising commercial carbon fibres as a source of exfoliated graphene oxide was recognised by R.T.O.

## Supporting information



Supporting Information

## Data Availability

The data that support the findings of this study are available in the supplementary material of this article.

## References

[1] A. Jiříčková , O. Jankovský , Z. Sofer , D. Sedmidubský , Materials (Basel) 2022, 15, 920. 35160865 10.3390/ma15030920 PMC8839209

[2] F. Nichols , S. Chen , Chem. Rec. 2020, 20, 1505. 32975907 10.1002/tcr.202000090

[3] B. Dąbrowski , A. Żuchowska , Z. Brzózka , Colloids Surf. B Biointerfaces 2023, 221, 112998. 36371926 10.1016/j.colsurfb.2022.112998

[4] H. Sachdeva , Green Process. Synth. 2020, 9, 515.

[5] R. K. Gupta , Z. A. Alahmed , F. Yakuphanoglu , Mater. Lett. 2013, 112, 75.

[6] K. Gong , Z. Pan , A. H. Korayem , L. Qiu , D. Li , F. Collins , C. M. Wang , W. H. Duan , J. Mater. Civil Eng. 2015, 27, A4014010.

[7] S. Zhang , H. Wang , J. Liu , C. Bao , Mater. Lett. 2020, 261, 127098.

[8] S. Abdelaal , E. K. Elmaghraby , A. M. Abdelhady , M. Youssf , A. M. Rashad , I. I. Bashter , A. I. Helal , Diamond Relat. Mater. 2020, 101, 107613.

[9] M. Inagaki , F. Kang , in Materials Science and Engineering of Carbon: Fundamentals (Second Edition), (Eds: M. Inagaki , F. Kang ), Butterworth‐Heinemann, Oxford, 2014, pp. 219–525.

[10] T.‐N. Tran , C. D. Mauro , A. Graillot , A. Mija , Macromolecules 2020, 53, 2526.

[11] M. Q. Jian , H. H. Xie , K. L. Xia , Y. Y. Zhang , in Industrial Applications of Carbon Nanotubes, (Eds: H. Peng , Q. Li , T. Chen ), Elsevier, Boston, 2017, pp. 433–476.

[12] Z. Li , J. Chu , C. Yang , S. Hao , M. A. Bissett , I. A. Kinloch , R. J. Young , Compos. Sci. Technol. 2018, 163, 116.

[13] W. S. Hummers , R. E. Offeman , J. Am. Chem. Soc. 1958, 80, 1339.

[14] H. Yu , B. Zhang , C. Bulin , R. Li , R. Xing , Sci. Rep. 2016, 6, 36143. 27808164 10.1038/srep36143 PMC5093679

[15] Q. Yu , L. Wei , X. Yang , C. Wang , J. Chen , H. Du , W. Shen , F. Kang , Z.‐H. Huang , Appl. Surf. Sci. 2022, 598, 153788.

[16] H. Gautneb , E. Tveten , Norges Geologiske undersøkelse 2000, 436, 67.

[17] M. Bhandari , I.‐W. Nam , Recycling 2024, 9, 17.

[18] J. B. Donnet , O. P. Bahl , R. C. Bansal , T. K. Wang , in Encyclopedia of Physical Science and Technology (Third Edition), (Eds.: R. A. Meyers ), Academic Press, New York, 2003, pp. 431–455.

[19] M. Endo , in The Society of Fiber Science and Technology, Springer Japan, Tokyo 2016, pp. 327.

[20] A. M. Pourrahimi , R. L. Andersson , K. Tjus , V. Ström , A. Björk , R. T. Olsson , Sustain. Energy Fuels 2019, 3, 2111.

[21] L. Pötschke , P. Huber , G. Stegschuster , S. Schriever , N. Kroppen , J. Schmatz , T. Gries , L. M. Blank , P. Farber , M. A. Rosenbaum , Front. Chem. Eng. 2022, 4, 765682.

[22] B. K. Birdsong , B. W. Hoogendoorn , F. Nilsson , R. L. Andersson , A. J. Capezza , M. S. Hedenqvist , S. Farris , A. Guerrero , R. T. Olsson , Nanoscale 2023, 15, 13037. 37492887 10.1039/d3nr01048a

[23] G. Panzarasa , G. Consolati , M. Scavini , M. Longhi , F. Quasso , J. Carbon Res. 2019, 5, 6.

[24] C. A. Love‐Baker , T. M. Harrell , F. Vautard , J. Klett , X. Li , Carbon 2024, 224, 119037.

[25] K. J. Putman , M. R. Rowles , N. A. Marks , C. de Tomas , J. W. Martin , I. Suarez‐Martinez , Carbon 2023, 209, 117965.

[26] M. P. Araújo , O. S. G. P. Soares , A. J. S. Fernandes , M. F. R. Pereira , C. Freire , RSC Adv. 2017, 7, 14290.

[27] S. Stankovich , D. A. Dikin , R. D. Piner , K. A. Kohlhaas , A. Kleinhammes , Y. Jia , Y. Wu , S. T. Nguyen , R. S. Ruoff , Carbon 2007, 45, 1558.

[28] X. Zheng , W. Chen , G. Wang , Y. Yu , S. Qin , J. Fang , F. Wang , X.‐A. Zhang , AIP Adv. 2015, 5, 057133.

[29] B. Humbert , A. Burneau , J. P. Gallas , J. C. Lavalley , J. Non‐Cryst. Solids 1992, 143, 75.

[30] A. Lerf , H. He , M. Forster , J. Klinowski , J. Phys. Chem. B 1998, 102, 4477.

[31] L. G. Guex , B. Sacchi , K. F. Peuvot , R. L. Andersson , A. M. Pourrahimi , V. Ström , S. Farris , R. T. Olsson , Nanoscale 2017, 9, 9562. 28664948 10.1039/c7nr02943h

[32] A. Ganguly , S. Sharma , P. Papakonstantinou , J. Hamilton , J. Phys. Chem. C 2011, 115, 17009.

[33] Z. Huang , H. Chen , L. Zhao , W. Fang , X. He , W. Li , P. Tian , Environ. Int. 2019, 126, 289. 30825747 10.1016/j.envint.2019.02.030

[34] A. Solak , A. Erkal‐Aytemur , M. Erdoğan , İ. Aşık , H. Ekşi , S. Jeon , Z. Üstündağ , J. Electrochem. Soc. 2014, 161, H696.

[35] D. A. Erdogan , M. Sevim , E. Kısa , D. B. Emiroglu , M. Karatok , E. I. Vovk , M. Bjerring , Ü. Akbey , Ö. Metin , E. Ozensoy , Top. Catal. 2016, 59, 1305.

[36] M. R. Biradar , N. Kumar , P. K. Pathak , S. V. Bhosale , S. V. Bhosale , R. R. Salunkhe , Energy Adv. 2024, 3, 574.

[37] V. V. Ivanovskaya , P. Wagner , A. Zobelli , I. Suarez‐Martinez , A. Yaya , C. P. Ewels , GraphITA , Graphene Edge Structures: Folding, Scrolling, Tubing, Rippling and Twisting, (Eds: L. Ottaviano , V. Morandi ), Springer, Berlin, Heidelberg 2011, pp. 75–85.

[38] J. D. Bernal , Proc. Royal Soc. London 1924, 106, 749.

[39] R. E. Franklin , Proc. Royal Soc. London. Series A. 1951, 209, 196

[40] H. Terrones , R. Lv , M. Terrones , M. S. Dresselhaus , Rep. Prog. Phys. 2012, 75, 062501. 22790648 10.1088/0034-4885/75/6/062501

[41] J. Cao , P. He , M. A. Mohammed , X. Zhao , R. J. Young , B. Derby , I. A. Kinloch , R. A. W. Dryfe , J. Am. Chem. Soc. 2017, 139, 17446. 29090921 10.1021/jacs.7b08515

[42] R. A. Rochman , S. Wahyuningsih , A. H. Ramelan , Q. A. Hanif , IOP Conf. Ser.: Mater. Sci. Eng. 2019, 509, 012119.

[43] B. D. Ossonon , D. Bélanger , RSC. Adv. 2017, 7, 27224.

[44] I. O. Faniyi , O. Fasakin , B. Olofinjana , A. S. Adekunle , T. V. Oluwasusi , M. A. Eleruja , E. O. B. Ajayi , SN Appl. Sci. 2019, 1, 1181.

[45] M. MInus , S. Kumar , JOM 2005, 57, 52.

[46] S. C. Bennett , D. J. Johnson , Soc. Chem. Ind. 1978, 1, 377.

[47] E. M. Pérez , N. Martín , Chem. Soc. Rev. 2015, 44, 6425. 26272196 10.1039/c5cs00578g

[48] C. A. Hunter , J. K. M. Sanders , J. Am. Chem. Soc. 1990, 112, 5525.

[49] K. Parvez , Z. S. Wu , R. Li , X. Liu , R. Graf , X. Feng , K. Müllen , J. Am. Chem. Soc. 2014, 136, 6083. 24684678 10.1021/ja5017156

[50] L. Zhao , J. Tang , M. Zhou , K. Shen , New Carbon Mater. 2022, 37, 544.

[51] K. Yokwana , B. Ntsendwana , A. N. Nxumalo , S. D. Mhlanga , J. Mater. Res. 2023, 38, 3239.

[52] Y. Zou , I. A. Kinloch , R. A. W. Dryfe , J. Mater. Chem. A. 2024, 2, 19495.

[53] U. Latif , M. A. Raza , Z. U. Rehman , J. Iqbal , N. Lee , S. M. Z. Mehdi , M. F. Maqsood , S. Hussain , Int. J. Energy Res. 2022, 46, 9643.

[54] S. Khaliha , A. Bianchi , A. Kovtun , F. Tunioli , A. Boschi , M. Zambianchi , D. Paci , L. Bocchi , S. Valsecchi , S. Polesello , A. Liscio , M. Bergamini , M. Brunetti , M. Luisa Navacchia , V. Palermo , M. Melucci , Sep. Purif. Technol. 2022, 300, 121826.

[55] M. N. Pervez , T. Jiang , J. K. Mahato , A. K. Ilango , Y. Kumaran , Y. Zuo , W. Zhang , H. Efstathiadis , J. I. Feldblyum , M. V. Yigit , Y. Liang , ACS ES&T Water 2024, 4, 2968. 39021580 10.1021/acsestwater.4c00187 PMC11249979

