# Supplementary material for: Making Synthetic 2D Graphene Oxide Nanosheets by Electrochemical Oxidation of Commercial Carbon Fibres
Source: Small. 2025 Feb 21;21(16):2408972. doi: 10.1002/smll.202408972 (PMC12019911; doi:10.1002/smll.202408972)
Supplement: Supplementary file 1 — Supporting Information [file SMLL-21-2408972-s001.pdf]

NANO · MICRO
small

Supporting Information

for *Small*, DOI 10.1002/smll.202408972

Making Synthetic 2D Graphene Oxide Nanosheets by Electrochemical Oxidation of Commercial Carbon Fibres

*Alba Español, Anton Bjurström, Björn Birdsong, Fritjof Nilsson, Annu Pandey, Xiaojun Ren, Rakesh Joshi, Stefano Farris and Richard T Olsson**

Supporting information

Making synthetic 2D Graphene oxide nanosheets by electrochemical oxidation of commercial carbon fibres

Alba Espanol[a], Anton Bjurström[a,b,c], Björn Birdsong[a], Fritjof Nilsson[a], Annu Pandey[a], Xiaojun Ren[d], Rakesh Joshi[d], Stefano Farris[e] and Richard T Olsson[a,b,*]

[a.] *Department of Fibre and polymer Technology, School of Chemical Science and Engineering, KTH Royal Institute of Technology, SE-106 91 Stockholm, Sweden.*
[b.] *Wallenberg Initiative Materials Science for Sustainability, Department of Fibre and Polymer Technology, KTH Royal Institute of Technology, Stockholm, SE-10691 Stockholm, Sweden.*
[c.] *NKT HV Cables, Technology Consulting, SE-721 78, Västerås, Sweden*
[d.] *School of Materials Science and Engineering, University of New South Wales, Australia*
[e.] *Department of Chemistry, University of Milan, via Golgi 19, 20133 Milan, Italy*
** Corresponding author: Richard T. Olsson (rols@kth.se)*

Table S1

Carbon source	Acid	Weight percentage	Potential	Results
CF	HNO_3	5%	5V	Oxidized carbon amorphous particles
			4V	Multilayer graphite oxide
			3V	Monolayer graphene oxide
			2V	No reaction
		1%	3V	No reaction
		10%	3V	Slight colouring after 45min
		50%	3V	Slight colouring after 2hours
	H_3PO_4	5%	3V	No colouring
			5V	Slight colouring after 1h30
		10%	3V	No colouring
			5N	No colouring
	Lactic	5%	3V	No reaction
			5V	No colouring
	H_2SO_3	5%	3V	No colouring
			5V	Slight colouring after 2h10
		10%	5V	Slight colouring after 4hours
		98.3% (conc.)	5V	No colouring
	Water	(+ Sodium chloride as electrolyte)	3V	No reaction
			5V	No reaction

† No reaction: No bubbling was observed on the anode, the reduction of H^+ into H_2(g) is therefore not occurring, no electrochemical reaction is occurring.

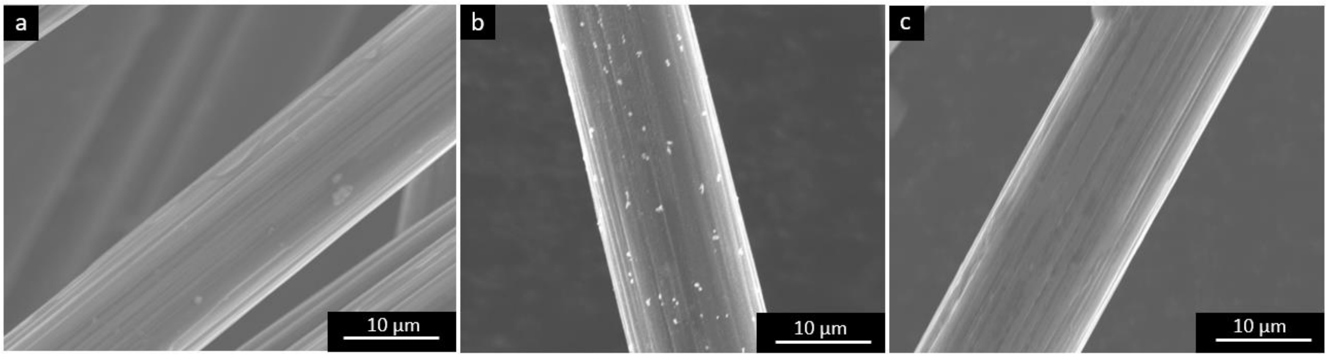

Fig **S1.** SEM micrographs of uncoated carbon fibres (a), High temperature treated fibres (b) and high voltage treated fibres (c).

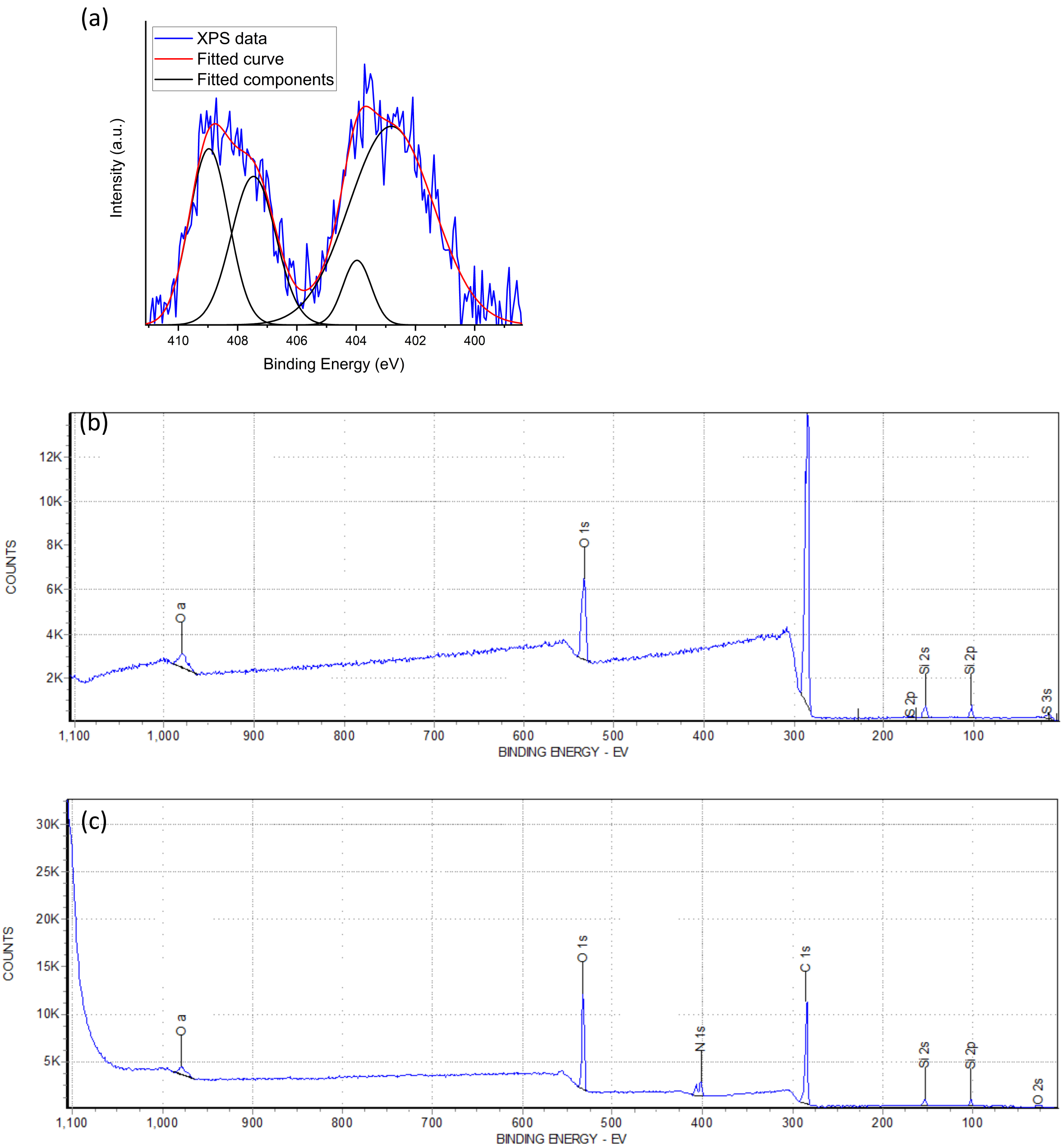

Fig S2. (a)N 1s XPS spectra of synthesised GO under 3V in 5 wt % nitric acid and total XPS spectra of (b) commercial GO and (c) synthesised GO**.**

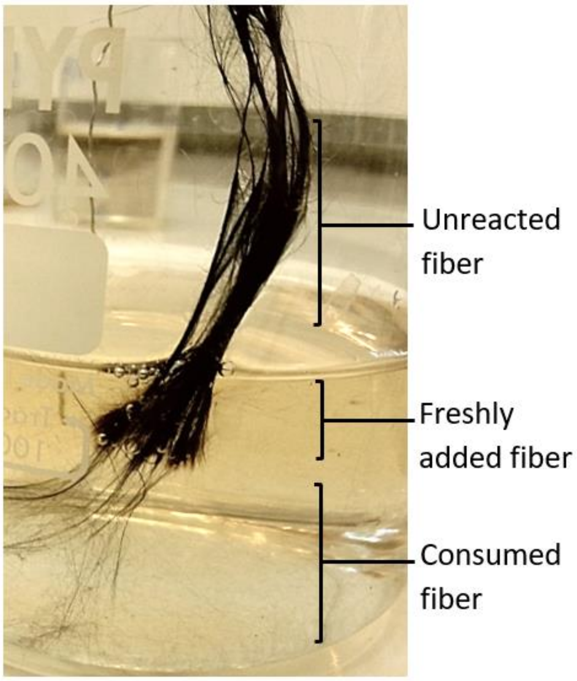

Fig S3. Carbon fibres under 5V potential illustrating the consuption of the fiber in the making of GO.

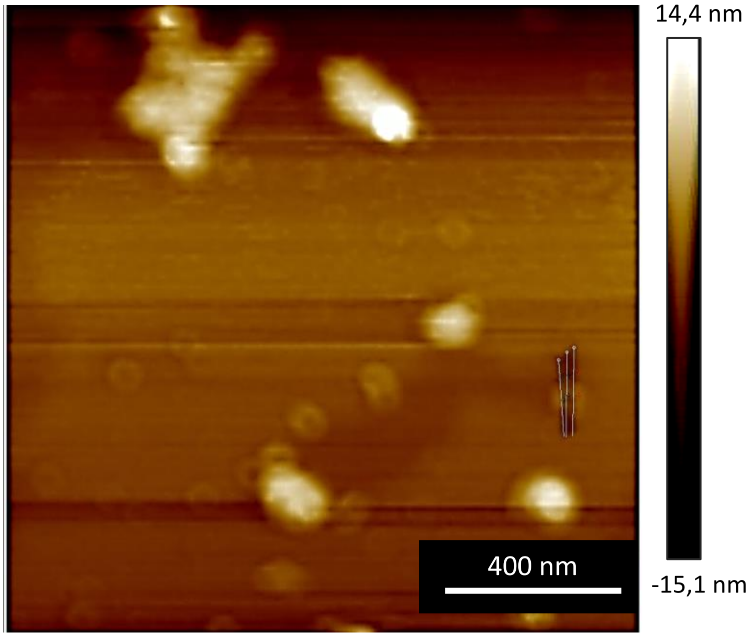

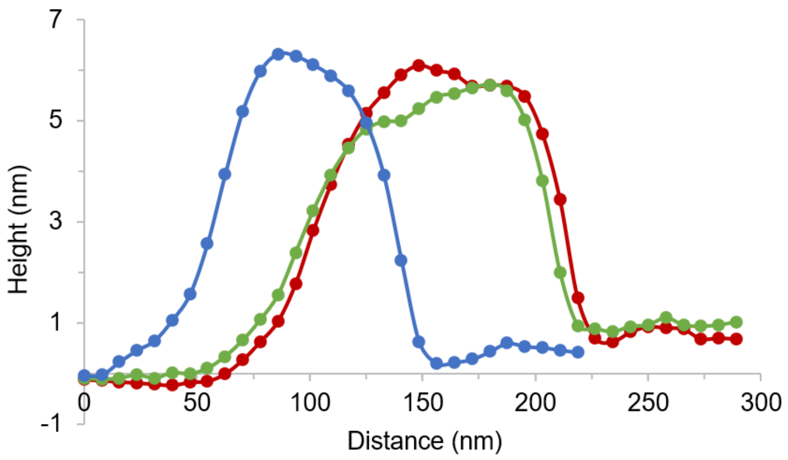

Fig S4. AFM image and height profile of particles synthesized under 4 Volts in 5wt% nitric acid and height profile

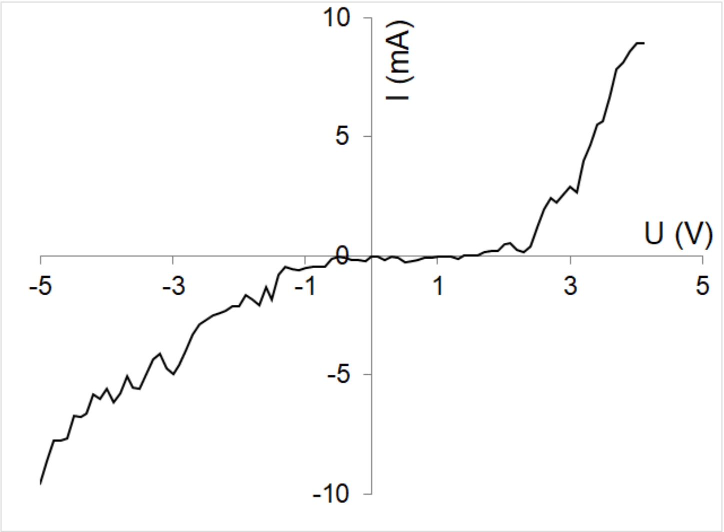

Fig S5. I-V curve (average of triplicate measurements) performed in 5% wt nitric acid.

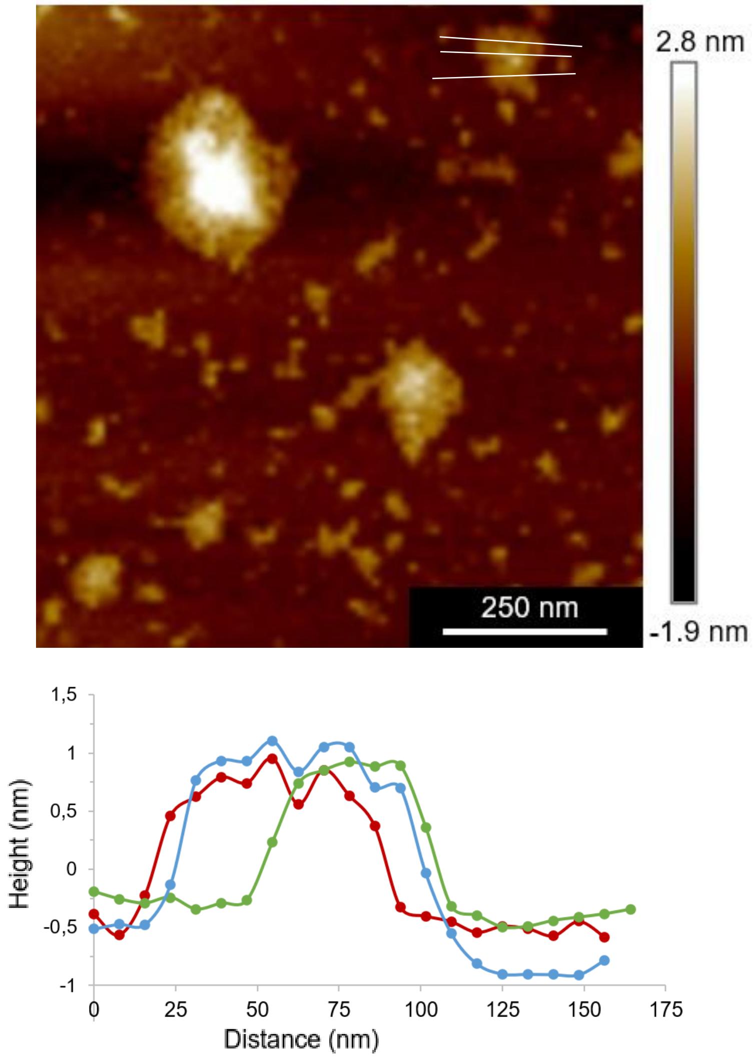

Fig S6. AFM image and height profile of scale-up synthesised sheets under 3v in 5 wt% nitric acid.

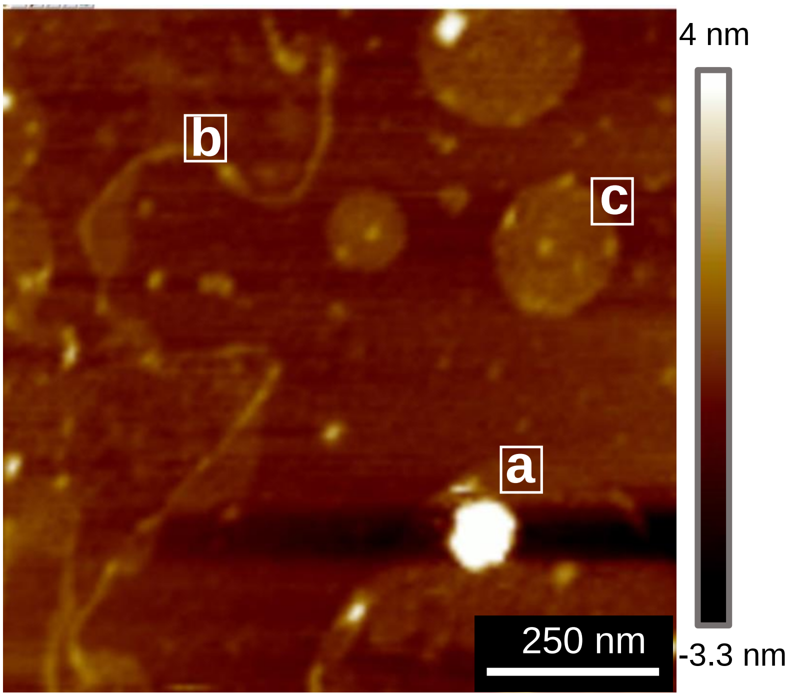

Fig S7. AFM image of synthesised GO sheets under 3v in 5 wt% nitric acid showing agglomerated graphene oxide sphere (a), fiber residue (b) and graphene oxide monolayer nanosheet (c).
